# Overexpression of *DDR1* contributes to gastric cancer progression by inhibiting the Hippo pathway

**DOI:** 10.7555/JBR.39.20250198

**Published:** 2025-06-03

**Authors:** Haiying Han, Tianqi Shen, Tingting Zhou, Yixuan Yang, Weiyi Toy, Yin Yin Choo, Fan Lin, Yoon Pin Lim

**Affiliations:** 1 Department of Nursing, School of Medicine, Hangzhou City University, Hangzhou, Zhejiang 310000, China; 2 Department of Cell Biology, School of Basic Medical Sciences; Institute for Brain Tumors & Key Laboratory of Rare Metabolic Diseases; The Affiliated Cancer Hospital, Nanjing Medical University, Nanjing, Jiangsu 211166, China; 3 Department of Cancer Biology and Innovation, Guoke Ningbo Life and Health Industry Research Institute, Ningbo, Zhejiang 315000, China; 4 Department of Biochemistry, Yong Loo Lin School of Medicine, National University of Singapore, Singapore 117545, Singapore; 5 NUS Graduate School of Integrative Sciences and Technology, Singapore 117456, Singapore

**Keywords:** iTRAQ, gastric cancer, *DDR1*, 7rh, Hippo pathway, phosphoproteomic analysis

## Abstract

Gastric cancer (GC) is a prevalent and devastating disease with a poor prognosis. The lack of biomarkers for early detection and effective targeted therapeutics for GC patients represents two major challenges. Through isobaric tags for relative and absolute quantitation (iTRAQ) coupled with liquid chromatography-tandem mass spectrometry (LC-MS/MS) phosphoproteomic analysis of 14 GC and gastric epithelial cell lines, we discovered the discoidin domain receptor tyrosine kinase 1 (DDR1) as a top potential drug target out of 40 tyrosine kinases detected along with over 1000 phosphoproteins profiled. The DDR1 protein and mRNA levels were upregulated in GC cells concurrent with *DDR1* gene amplification. Immunohistochemistry staining of more than 200 clinical samples revealed that DDR1 was overexpressed in approximately 41% and 48% of the intestinal and diffuse types of GC cases, respectively, compared with only 3.5% in normal tissues. Higher *DDR1* expression was associated with poor prognosis. In cellular models, *DDR1* overexpression led to accelerated proliferation, invasion, and malignant transformation, putatively *via* inhibition of the Hippo pathway and consequent activation of YAP-TEAD target gene expression. Notably, *DDR1*-overexpressing GC cells exhibited high vulnerability to selective DDR1 inhibitors. The present study provides preclinical support for the application of DDR1-selective inhibitors in *DDR1*-overexpressing GC.

## Introduction

Gastric cancer (GC) is the fifth most prevalent cancer and the fifth leading cause of cancer-related deaths worldwide, affecting more than 660000 individuals in 2022^[1]^. The prognosis of GC remains poor, especially in Asia^[2]^. The lack of sensitive and specific biomarkers and effective treatments tailored for GC patients represents two major challenges in the management of this disease.

Tyrosine kinases are a particularly interesting group of proteins. Although there are about 100 members in the tyrosine kinase family, over half have been linked to human cancers^[3]^, making them attractive drug targets. Numerous tyrosine kinase inhibitors (TKIs) are either approved by the Food and Drug Administration or are undergoing various stages of clinical trials. Discoidin domain receptor (DDR), a receptor tyrosine kinase (RTK), exhibits an extracellular domain with strong homology to lectin discoidin Ⅰ, a protein secreted by the slime mold *Dictyostelium discoideum*^[4]^. As one of the two members of the DDR subclass, DDR1 may be activated by collagen Ⅰ–Ⅴ, but its ligand-induced activation mechanism remains unique and less well understood than that of other RTKs^[5]^. *DDR1* is frequently overexpressed in various types of cancers, including gastric^[6]^, breast, lung, brain, liver, esophageal cancer, and leukemia^[7]^. Growing evidence indicates that *DDR1* is pivotal in cancer progression. To date, no DDR1-targeting drugs have received clinical approval^[8]^.

Multi-target kinase inhibitors like dasatinib have been shown to effectively inhibit *DDR2* gain-of-function mutations, which account for approximately 4% of lung squamous cell carcinomas^[9]^. This was the first report demonstrating the usefulness of pharmacological inhibition of the DDR family member in cancer treatment. Targeting DDR is further supported by the discovery of several DDR1 pharmacological inhibitors^[10–12]^. For example, two selective DDR1 inhibitors, 7rh and 7rj, exhibited potent anti-proliferative effects and strong suppression of invasion, adhesion, and tumorigenicity in cancer cells expressing high levels of DDR1^[10]^. Further investigations are needed to validate DDR1 as a viable drug target. Notably, the primary oncogenic signaling pathway linked to DDR1 remains unclear, particularly in GC. Emerging evidence implicates dysregulation of the Hippo signaling pathway in gastric carcinogenesis^[13]^. The Hippo pathway, regulated by the MST1/2-LATS1/2 kinase cascade, phosphorylates YAP/TAZ to prevent their nuclear accumulation and TEAD-mediated oncogenesis. In the present study, we investigated the association between DDR1 and the Hippo pathway in GC.

The cancer phosphoproteome is a rich resource from which biomarkers and drug targets can be mined. Phosphoproteomics focuses on targets that are regulated by phosphorylation, thus implying that the detected proteins are likely to be key regulatory factors in cancer. It has been instrumental in identifying novel oncogenes, oncogenic mutations, and kinase substrates^[14]^. In the present study, phosphoproteomic analysis of a large panel of GC cell lines identified DDR1 overexpression. We subsequently investigated the clinical relevance, oncogenic role, and efficacy of DDR1 inhibitors in GC, as well as the association between DDR1 and the Hippo pathway.

## Materials and methods

### Cell culture

All GC cell lines were obtained from the American Type Culture Collection (Manassas, VA, USA) and tested for *Mycoplasma* contamination before experimentation. Cells were cultured in RPMI-1640 (Thermo Fisher Scientific Hyclone, Logan, UT, USA) supplemented with 10% fetal bovine serum (Hyclone) and 1% penicillin-streptomycin (Biological Industries, Beit Haemek, Israel), which served as the basal medium for all cell types except HFE145 and 293T. HFE145 and 293T cells were cultured in DMEM (Hyclone) following established protocols. Cell cultures were maintained at 37 ℃ in a 5% CO_2_ humidified atmosphere with strict passage control (< 30 population doublings) to ensure experimental consistency throughout subsequent analyses.

### Phosphoprotein affinity purification, isobaric tags for relative and absolute quantitation (iTRAQ) labeling, and mass spectrometry

In brief, total protein was isolated from cultured cells using RIPA buffer (Beyotime, Shanghai, China) supplemented with a protease inhibitor cocktail (Beyotime), and the lysates were centrifuged at 4 ℃ at 16000 *g* for 15 min. Sixty milligrams of total protein were purified using immunoaffinity techniques with 4G10 anti-phosphotyrosine antibodies. The purified phosphoproteins underwent denaturation, cysteine blocking, and 8-plex iTRAQ labeling (Applied Biosystems, Foster City, CA, USA). Details for mass spectrometry analysis of the iTRAQ-labeled peptides are described in ***Supplementary Data*** (available online).

### Immunohistochemistry (IHC) staining

Clinical specimens were collected from the National University Hospital (Singapore) with Institutional Review Board approval (Approval No. NUS-IRB REFERENCE CODE:08-347), with accompanying histopathology reports. Tissue microarray and individual tissue sections were subjected to dewaxing, antigen retrieval, quenching, blocking, and incubation with DDR1 antibody (Cat. #5583, Cell Signaling Technology, Danvers, MA, USA) at a 1∶500 dilution in a humidified chamber maintained at 4 ℃ for 16 h. After detection, all IHC stains were scored from zero to three according to the stain intensity (0: no staining; 1+: mild; 2+: moderate; and 3+: strong staining). Moreover, cells with a DDR1-staining score greater than one were defined as DDR1-positive. Details of these methods are described in ***Supplementary Data***.

### Western blotting and immunoprecipitation

Immunoblotting and immunoprecipitation were performed as previously described^[15]^. In brief, cells were washed with ice-cold PBS and then lysed using an NP-40 lysis buffer (150 mmol/L NaCl, 50 mmol/L Tris, pH 7.4, 0.5% Nonidet-P40, 0.5% Triton X-100, 1 mmol/L EDTA) with a protease and phosphatase inhibitor cocktail (Pierce, Rockford, IL, USA). After vortexing, cell lysates were centrifuged at 16000 *g* for 20 min at 4 ℃. Thirty to fifty micrograms of proteins were separated by SDS-PAGE (10% acrylamide gel). The proteins were then transferred onto PVDF membranes (Pierce). Following blocking, the blots were initially incubated with primary antibodies overnight at 4 ℃, followed by anti-mouse/rabbit IgG-HRP conjugates (Pierce) at room temperature for one hour with reciprocal shaking, with subsequent detection using ECL HRP substrate.

Clarified lysates (0.5–1 mg) were incubated with specific antibodies (1–5 μg) at 4 ℃ for 16 (± 0.5) h. Magnetic separation was performed using Dynabeads Protein G (Invitrogen, Carlsbad, CA, USA) at room temperature for 15–20 min. Immunoprecipitates were thoroughly washed four times (5 min each) in buffer containing 1 mmol/L EDTA, 50 mmol/L Tris (pH 7.5), 0.5% Nonidet P-40, 150 mmol/L NaCl, 1 mmol/L Na_3_VO_4_, 10% glycerol, and 0.5% Triton X-100. Cell lysis and Western blotting analyses were performed as described above.

### *DDR1* overexpression and knockdown

siRNA oligonucleotide sequences (Invitrogen) are as follows: non-silencing siRNA, 5*'*-CGUACGCGGAAUACUUCGA-3*'*; *DDR1* siRNA1, 5*'*-UGUGCAUGUUGUUACAGUGGACCUG-3*'*; *DDR1* siRNA2, 5*'*-AGAUCUUGACAGCUACCAGCAAAGG-3*'*. Three *DDR1* shRNA sequences (Thermo Fisher Scientific Pierce) are as follows: V2LHS_84433, 5*'*-TGTTGTTACAGTGGACCTG-3*'* (*DDR1*sh1); V2LHS_202770, 5*'*-TGTTGATGAGGATAGTGTC-3*'* (*DDR1*sh2); V3LHS_392923, 5*'*-CGTTGTAGATCCACCTGCA-3*'* (*DDR1*sh3). For *DDR1* overexpression experiments, a plasmid pLenti-CMV-Puro-*DDR1*-Flag was constructed. Transfection and transduction protocols for *DDR1* knockdown and overexpression in HFE145 and GC cells were performed as previously described^[15]^. Details about these methods are described in ***Supplementary Data***.

### Cell proliferation and colony formation assays

Cell proliferation and anti-proliferation experiments were performed using MTS Reagent obtained from Promega (Madison, WI, USA). Briefly, 2000 cells per well were seeded in 96-well plates on day 0 and treated with various concentrations of DDR1 inhibitors for three days. The absorbance of the cell culture medium after incubation with 20 μL MTS reagent was measured using a plate reader (Varioskan Flash, Thermo Fisher Scientific, Waltham, MA, USA) at 490 nm on days 1–5.

In the colony formation assay, 400 cells per well were seeded in 6-well plates and treated with various concentrations of DDR1 inhibitors (7rh^[10]^ and DDR1-in-1^[11]^). Once the control wells reached confluency, all cells were fixed and stained with a solution containing 6% (V/V) glutaraldehyde and 0.5% (m/V) crystal violet (Sigma). The plates were then imaged using a Chemi-Doc MP Imaging System (Bio-Rad). Experiments were carried out with a minimum of three technical replicates and across three independent biological experiments.

### Dual luciferase reporter assay

The transcriptional regulatory interaction between DDR1 and YAP-TEAD was assessed using the Dual-Luciferase Reporter Assay System (Promega) and quantified using a Luminoskan Ascent microplate luminometer (Thermo Fisher Scientific). Firefly luciferase signals were normalized to Renilla luciferase signals.

### Real-time reverse transcription PCR (RT-qPCR)

Total RNA was extracted using the Qiagen RNasy isolation kit (Qiagen, Venlo, Netherlands) according to the manufacturer's protocol. First-strand cDNA was synthesized using a High-Capacity cDNA Reverse Transcription Kit (Thermo Fisher Scientific). Transcript abundance was quantified by real-time PCR using the QuantiFast Probe PCR kit (Qiagen). Pre-designed primers were obtained from IDT (Coralville, Iowa, USA). *DDR1* primers: forward, 5*'*-AAGGGACATTTTGATCCTGCC-3*'*, *T*_m_ = 60.3 ℃; reverse, 5*'*-CCTTGGGAAACACCGACCC-3*'*, *T*_m_ = 62.6 ℃.

### Xenograft drug intervention studies

All animal experiments were approved by the Institutional Animal Care and Use Committee at the National University of Singapore (Approved IACUC Protocol No: 085-12) and conducted in accordance with the institutional guidelines. Mice were housed and maintained in accordance with the institutional guidelines, adhering to Singaporean regulations. A total of 5 × 10^6^ NUGC3_Luc_NS shRNA (control) and NUGC3_Luc_*DDR1* shRNA (*DDR1* knockdown) cells were inoculated subcutaneously into eight-week-old female SCID mice (InVivos, Singapore). Neoplastic progression was quantified *via* bioluminescence using the IVIS 200 imaging system (Xenogen, CA, USA). The drug was formulated as previously described^[10]^. Briefly, 7rh was dissolved in mixed solvents (H_2_O∶Cremophor EL∶ethanol∶dimethyl sulfoxide = 90∶4∶4∶2) to obtain a clear solution. This yielded a solution with a concentration of 2.5 mg/mL and was administered to mice at a dose of 25 mg/kg daily for two weeks by oral gavage. All mice were uniformly euthanized when the maximum tumor volume (2000 mm^3^) was reached.

### Statistical analysis

The figures show *in vitro* data as the mean ± standard deviation, while *in vivo* tumor growth data are displayed as the mean ± standard error of the mean. In all *in vitro* experiments, Student's *t*-test was applied to identify statistical significance among groups and treatments. A *P*-value of less than 0.05 was considered statistically significant.

## Results

### iTRAQ-based phosphoproteomics revealed DDR1 to be a major tyrosine kinase that is aberrantly expressed in GC cells

**Figure 1:**
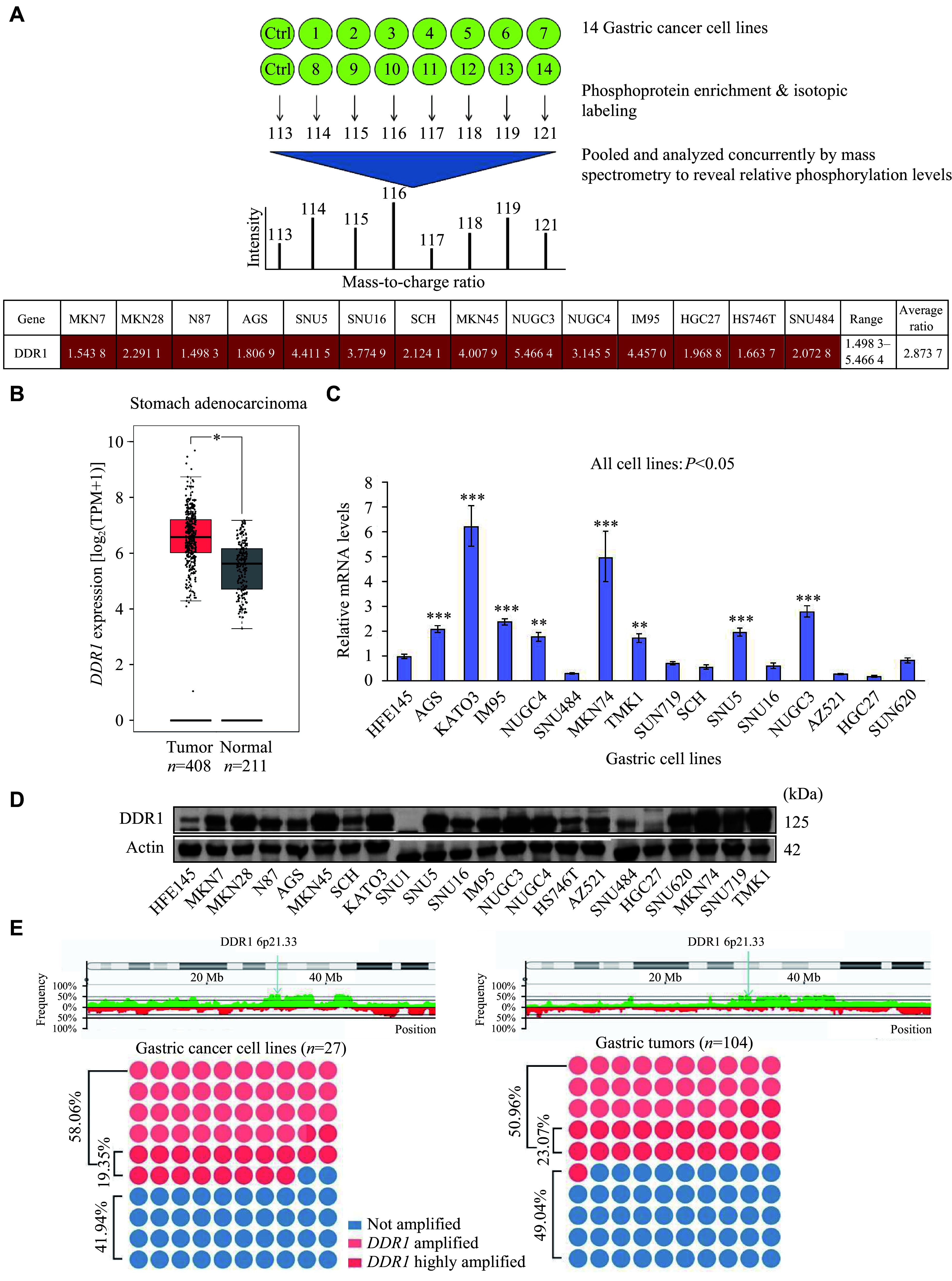
Discovery of *DDR1* overexpression in gastric cancer (GC) cell lines. A: Aberrant upregulation of DDR1 phosphorylation in GC cells was identified by an isobaric tags for relative and absolute quantitation (iTRAQ)-based phosphoproteomic screen. Upper: schematic workflow of phosphoproteomic profiling of proteins in non-cancer and GC cells. Lower: fold change, range, and average ratio of DDR1 in various GC cell lines relative to HFE145 cells as the control. B: Box plot analysis of *DDR1* expression in GC tissues versus adjacent normal tissues from TCGA and GTEx databases. ^*^P < 0.05. C and D: Validation of mRNA levels and protein expressions of DDR1 by real-time reverse transcription PCR analysis (C) and Western blotting (D) in HFE145 and GC cell lines. ^**^P < 0.01 and ^***^P < 0.001, compared the *DDR1* mRNA levels in non-cancer cell line HFE145 with other GC cell lines by Student's *t*-test. E: Array comparative genomic hybridization analysis of *DDR1* copy number alteration in 27 GC cell lines and 104 GC samples, and the percentages of *DDR1* amplified or highly amplified cases compared with HFE145 and normal samples.

Phosphoproteomics was conducted to map molecular variations in GC and identify novel GC-associated proteins, which permit the enrichment and detection of low-abundance signaling proteins that are otherwise not captured by shotgun proteomics. Briefly, the phosphotyrosine proteomes of a panel of 14 GC cell lines (MKN7, MKN45, NCI-N87, IM95, SCH, SNU16, SNU5, MKN28, NUGC3, NUGC4, AGS, HGC27, HS746T, and SNU484) were compared with that of the non-cancer cell line HFE145 using the 4G10 anti-phosphotyrosine antibody affinity purification combined with iTRAQ and electrospray tandem mass spectrometry^[16]^. A flowchart of the experimental study design is shown in ***Fig. 1A***. More than 1000 phosphoproteins were detected and relatively quantified across 15 cell lines with non-cancer cell line HFE145 as the denominator^[17]^. DDR1 was one of the top hits among nearly 40 tyrosine kinases detected. The levels of phosphorylated DDR1 receptor tyrosine kinase were elevated in all 14 GC cell lines compared with HFE145, with an average overexpression of 2.87-fold (***Fig. 1A***). DDR1 was chosen for further studies because data regarding its role in GC remain scarce, despite some recent studies reporting its role and mechanism in GC progression^[18]^.

Subsequently, analysis of *DDR1* expression in GC tissues through GEPIA2 (http://gepia2.cancer-pku.cn), which integrates GTEx and TCGA databases, revealed significantly elevated levels in tumor tissues, with *P* < 0.05 and |log_2_(fold change)| ≥ 0.5 as significance thresholds (***Fig. 1B***). Next, we analyzed the *DDR1* gene at the mRNA, protein, and DNA levels to explore the molecular cause of elevated phosphorylated DDR1. RT-qPCR analysis revealed that 8/15 (53%) GC cell lines had higher *DDR1* mRNA levels than HFE145 (***Fig. 1C***). Western blotting revealed that more than 86% (18/21) of the GC cell lines overexpressed DDR1 compared with HFE145 (***Fig. 1D***). Array comparative genomic hybridization analysis showed *DDR1* amplification in 50.96% of 104 gastric tumors and 58.06% of 27 gastric cell lines (***Fig. 1E***). The discordance between gene copy number and transcript and protein levels suggests that while genomic amplification and transcription may account for overexpression of DDR1, post-transcriptional and translational regulation is likely to be involved as well.

### *DDR1* was overexpressed in diffuse and intestinal types of GC and was associated with poor prognosis

**Figure 2:**
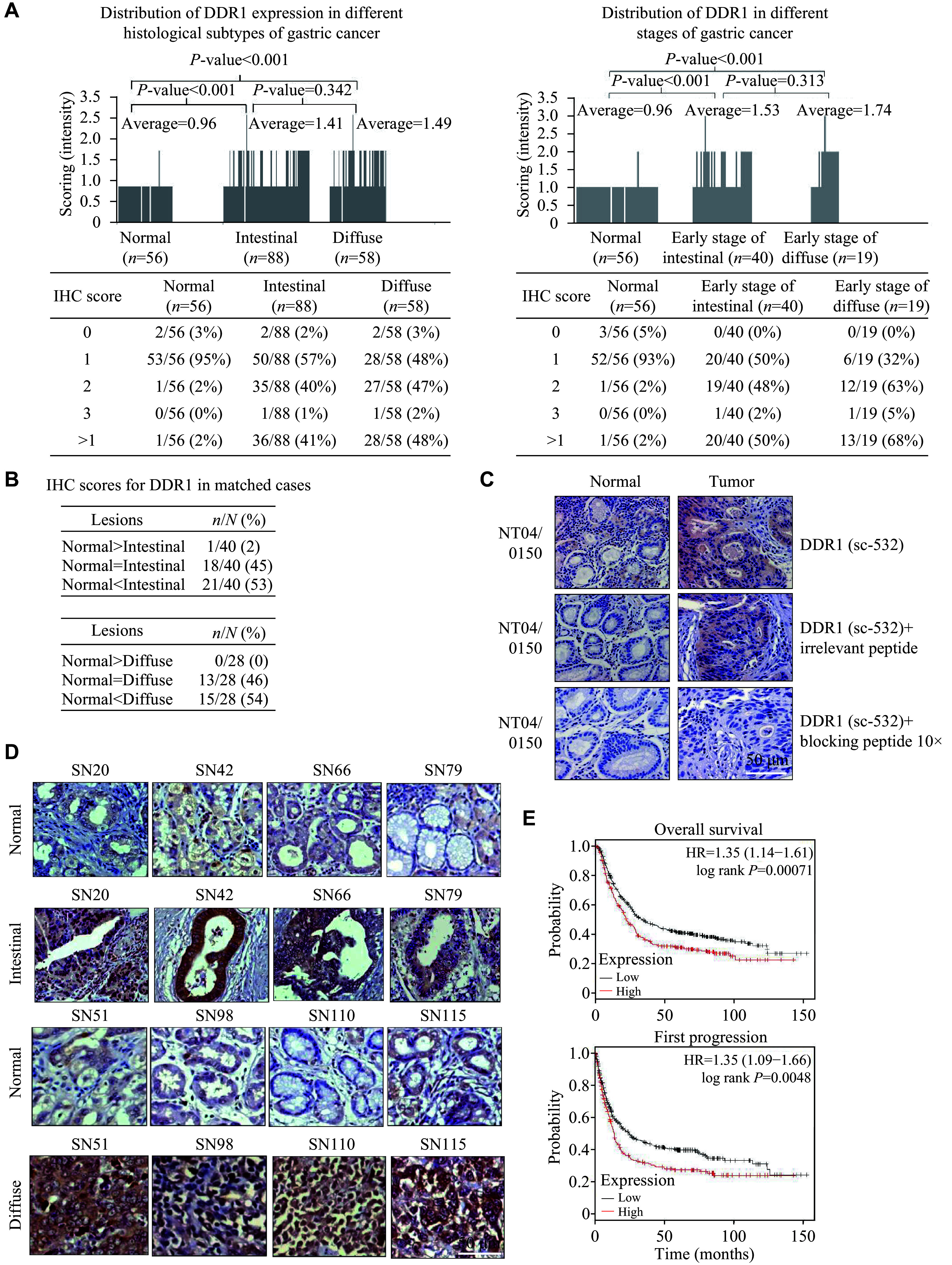
Overexpression of *DDR1* in gastric tumor tissues. A: A summary of tissue microarray analysis of clinical gastric samples. A total of 146 cancer tissues and 56 normal tissues were analyzed. In the left panel, DDR1 IHC scores for the individual intestinal and diffuse types of gastric cancer (GC) samples were compared with those of normal samples. In the right panel, the same was done except that only samples from early-stage intestinal and diffuse GC were used in comparison to non-cancer tissue. B: IHC scores for DDR1 in matched normal and cancer tissues were compared. C: Peptide competition demonstrating the specificity of DDR1 antibody (sc-532) in IHC. Normal and gastric tumor tissue specimens were stained with the DDR1 antibody (sc-532) (upper) in the presence of either an irrelevant peptide (CEP68; middle) or a DDR1-specific peptide (lower). Scale bar = 50 μm. D: Representative IHC images (10× magnification) of DDR1 in four matched GC and normal tissues for intestinal (upper) and diffuse subtypes (lower). Scale bar = 50 μm. E: Kaplan–Meier plots for overall survival and first progression of patients segregated into *DDR1*-high and *DDR1*-low subgroups.

To assess the significance of DDR1 in clinical GC, we performed IHC staining of DDR1 in more than 200 specimens. The IHC scores for each sample were plotted as bar charts. ***Fig. 2A*** (left) shows that approximately 41% and 48% of the intestinal and diffuse types of GC had DDR1 IHC scores of > 1, respectively, compared with only approximately 2% in normal tissues. The average DDR1 IHC scores for normal, intestinal, and diffuse GC tissues were 0.96, 1.41, and 1.49, respectively. Further analysis of the correlation between DDR1 expression and the GC stage revealed that aberrant DDR1 expression occurred during early cancer development (***Fig. 2A***, right). Examination of the matched samples (*i.e.*, normal and tumor tissues from the same patients) showed that > 50% of the cases had DDR1 upregulated in tumor tissues compared with matched normal tissues, suggesting that DDR1 is required for cancer development (***Fig. 2B***). The DDR1 antibody specificity was tested by incubating the DDR1 antibody with a DDR1-derived peptide or a non-specific peptide derived from another protein before IHC. The results in ***Fig. 2C*** showed that the IHC signal generated by the DDR1 antibody was blocked by the DDR1-derived peptide but not by the non-specific control peptide. These results demonstrate that the IHC signal was specific to the DDR1 protein. ***Fig. 2D*** shows representative images of DDR1 expression in matched clinical gastric tissues.

To determine whether *DDR1* expression is associated with patient outcomes, we performed Kaplan–Meier (KM) analysis using the KM Plotter (http://kmplot.com/analysis), which assesses the effect of candidate genes on survival in 1065 GC patients^[19]^. The background database was compiled manually, incorporating gene expression data and details on relapse-free and overall survival from TCGA, EGA, and GEO (exclusively Affymetrix microarrays). To analyze the prognostic value of the *DDR1* gene, the patient samples were divided into two groups based on the median value of the dataset. The analysis did not impose restrictions on TNM, Lauren classification, sex, or treatment. Data from all probe sets available were used (210749_x_at, 207169_x_at, 1007_s_at, and 208779_x_at). The results indicated that *DDR1* expression was significantly associated with poorer overall survival (OS) and first progression (FP), with hazard ratios ranging from 1.14 to 1.61 for FP and 1.09 to 1.66 for OS. Representative KM plots for OS and FP using one of the gene probes are shown (***Fig. 2E***). Collectively, *DDR1* was overexpressed in GC, and its expression was associated with poor prognosis. The data support the notion that DDR1 is a viable drug target for GC therapy.

### *DDR1* played a critical role in gastric cell proliferation, malignant transformation, and invasion

**Figure 3:**
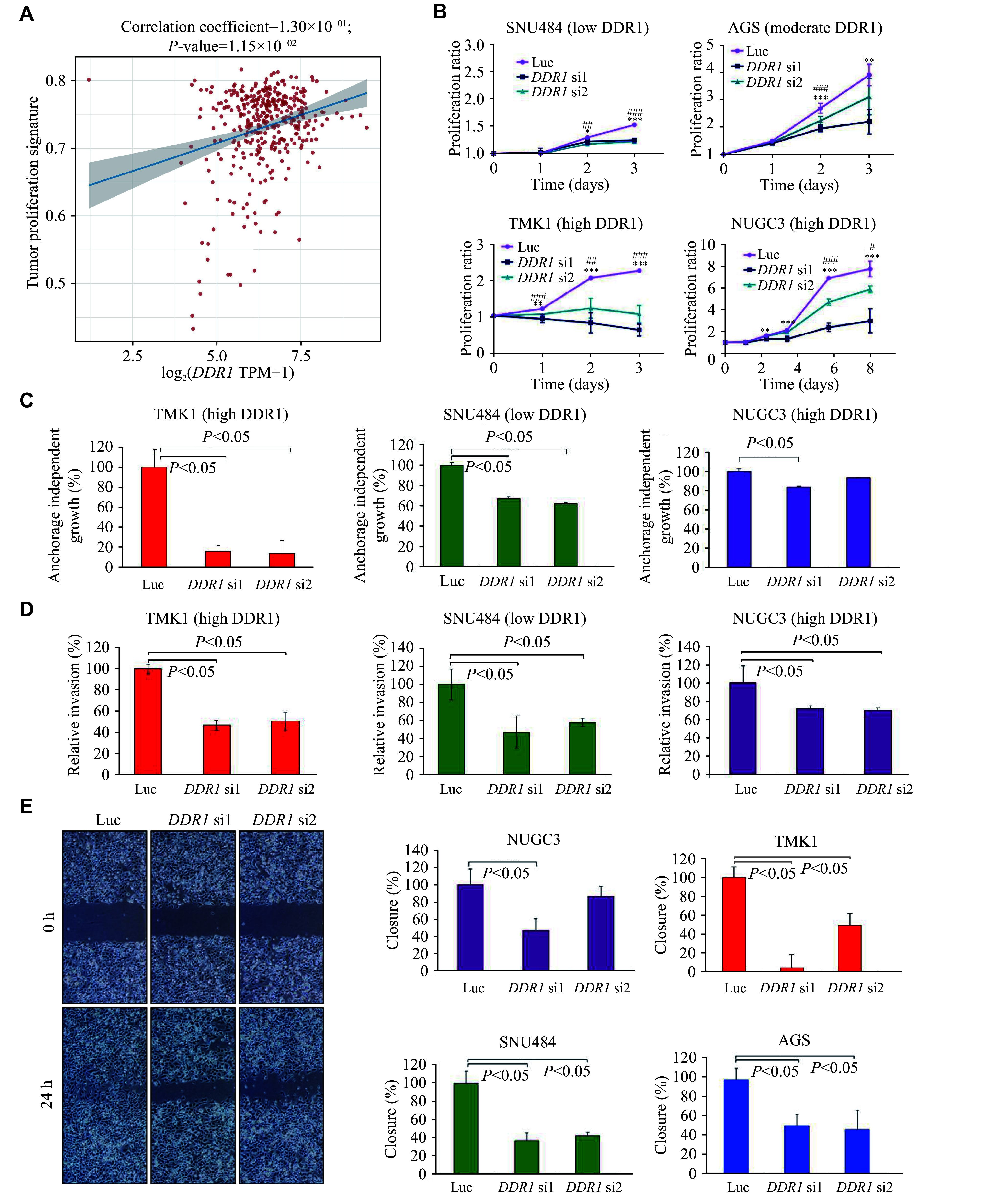
*DDR1* expression affected gastric cancer (GC) cell proliferation, growth, and invasion. A: Correlation between *DDR1* expression and tumor proliferation-associated gene set enrichment scores in TCGA GC patients. B–E: Proliferation curves (B), anchorage-independent growth (C), matrigel invasion (D), and wound healing (E) of GC cells transfected with either Luc siRNA or two *DDR1* siRNAs (*DDR1* si1 and *DDR1* si2). Representative microscopic images of wound healing experiments on SNU484 cells are shown (E left). Error bars represent mean ± standard deviation (*n* = 3). ^*/#^*P* < 0.05, ^**/##^*P* < 0.01, and ^***/###^*P* < 0.001, compared the *DDR1* si1 and *DDR1* si2 proliferation ratio with the control (Luc) group by Student's *t*-test.

The functional role of DDR1 in GC was examined to evaluate its potential as a drug target. We used gene sets from relevant pathways^[20]^ and performed single-sample Gene Set Enrichment Analysis (ssGSEA) to calculate pathway enrichment scores for each specimen, thereby establishing associations between biological samples and pathway activities. Subsequent correlation analysis revealed a significant positive correlation between *DDR1* expression levels and tumor proliferation signature scores in TCGA GC patients (***Fig. 3A***). *DDR1* knockdown significantly reduced the proliferation potential of multiple GC cell lines (***Fig. 3B; Supplementary Fig. 1*** upper [available online]). Based on protein enrichment ratios in ***Fig. 1A***, cell lines expressing DDR1 with a fold change of < 1.2, between 1.2 and 2.5, and > 2.5 relative to the control HFE145 cells were accordingly defined as low, moderate, and high, respectively. As a result, the DDR1-high cell lines appeared to be more affected by *DDR1*-specific siRNAs than the DDR1-low cell lines. *DDR1* knockdown also significantly reduced anchorage-independent growth of GC cells in soft agar, suggesting that *DDR1* is involved in the malignant transformation of gastric cells (***Fig. 3C***). Moreover, Matrigel invasion and wound healing assays revealed that *DDR1* knockdown significantly reduced the invasion and migration capacities of GC cells (***Fig. 3D*** and ***3E***; ***Supplementary Fig. 1*** lower [available online]), indicating that *DDR1* is required for invasion and migration to different extents in GC cells to varying extents.

Next, we generated three gastric cell lines overexpressing exogenous DDR1 to study the gain-of-function effect of *DDR1* by transducing HFE145, AGS, and MKN7 cells with lentiviral *DDR1* cDNA. As shown in ***Supplementary Fig. 2 ***(available online), the proliferation of the *DDR1* stable transfectants was significantly increased compared with the vector control and wild-type cells. The knockdown and overexpression of *DDR1* in the above experiments were validated by Western blotting relative to control cells in the respective cell lines (***Supplementary Fig. 3***, available online).

### Overexpression of *DDR1* inhibited the Hippo pathway and activated YAP-TEAD target gene expression

Although the involvement of DDR1 in inhibiting the Hippo pathway to enhance hepatocellular carcinoma cell stemness has been documented^[21]^, its role in regulating the Hippo pathway in GC remains unclear, despite various proposed DDR1 downstream pathways^[22]^. The Hippo pathway is involved in restraining cell proliferation and controlling organ size, and it has been found to be increasingly significant in various human cancers^[23–24]^. Our results showed that *DDR1* knockdown led to a concomitant reduction in total YAP and an increase in phospho-YAP levels in NUGC3 cells (***Fig. 4A***). Consistent with this result, *DDR1* knockdown led to a significant reduction in the YAP-TEAD reporter activity (***Fig. 4B***) and the expression levels of YAP-TEAD target genes such as *CTGF* and *CYR61* (***Fig. 4C***). Next, we demonstrated this finding *via* a *DDR1* gain-of-function model. Consistently, *DDR1* overexpression in AGS cells that intrinsically express moderate levels of DDR1 significantly increased total YAP expression and reduced the levels of phospho-YAP (***Fig. 4D***). *DDR1* overexpression also increased YAP-TEAD reporter activity (***Fig. 4E***) and the expression of *CTGF* and *CYR61* (***Fig. 4F***), whereas *DDR1* knockdown in the parental and *DDR1*-overexpressing AGS cells caused a significant reduction in YAP-TEAD reporter activity (***Fig. 4E***).

**Figure 4:**
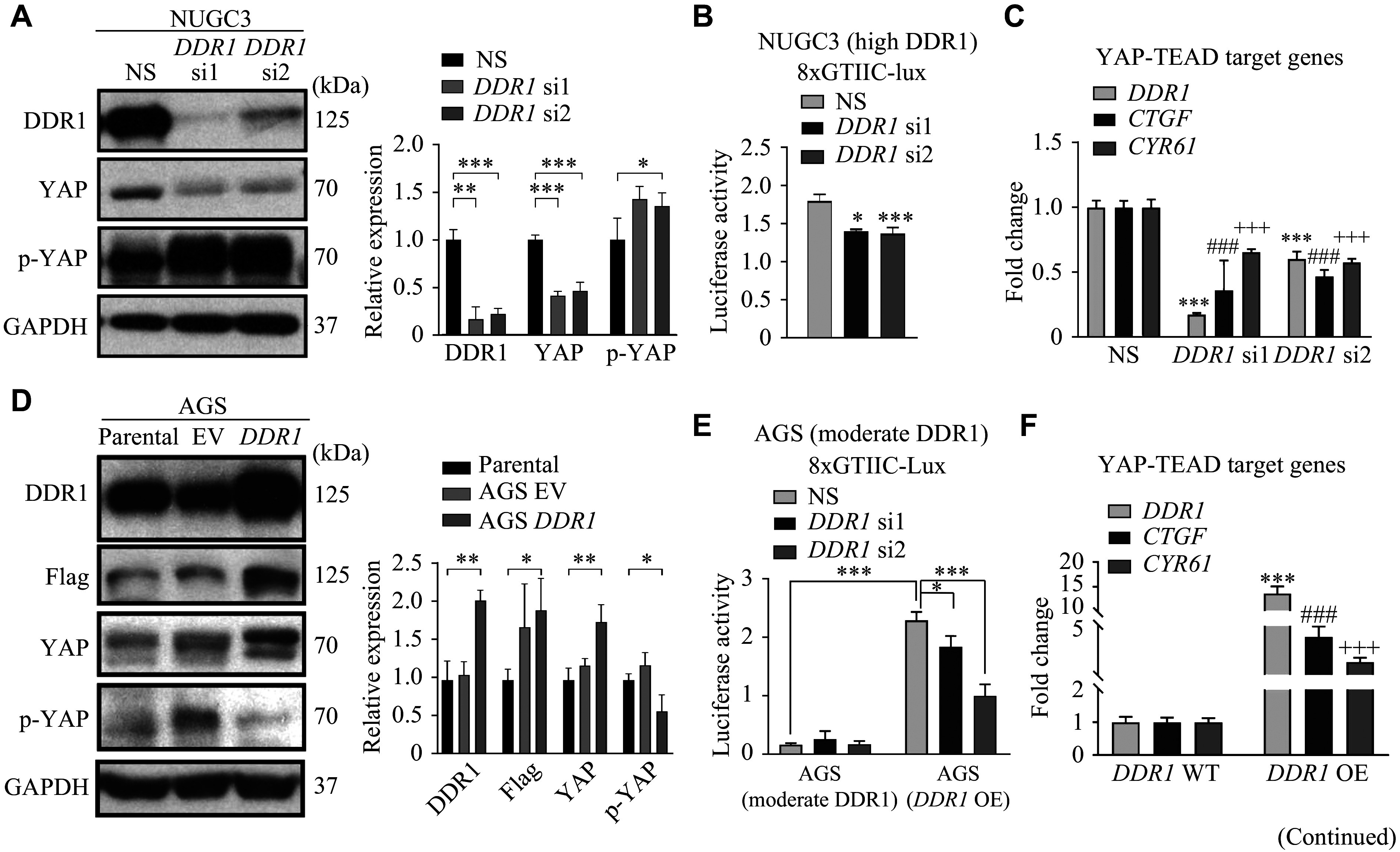


To further investigate the DDR1-Hippo pathway interaction, we also analyzed associated alterations in Hippo pathway components. We found that overexpression of *DDR1* caused the downregulation of YAP/TAZ, and *DDR1* knockdown increased the phosphorylation of YAP as well as several important regulators in the Hippo pathway, such as MST1 and SAV1. These results indicate a negative correlation between DDR1 and the Hippo pathway (***Supplementary Fig. 3***).

To obtain more evidence of the association between DDR1 and YAP in GC tissues, we selected 92 GC samples with positive YAP and DDR1 IHC staining and analyzed their correlation. The cytoplasmic and nuclear YAP total score was calculated by multiplying its IHC signal intensity by the percentage of tissue section stained positive for IHC (***Supplementary Fig. 4***, available online). There was a clear trend showing that nuclear YAP was higher in the DDR1 high (score = 2, 3) than in the DDR1 low (score = 0, 1) group (median: 0.3 *vs.* 0.8; mean: 0.52 *vs.* 0.71), while the difference in cytoplasmic YAP between the two groups was only marginal (median: 0.2 *vs.* 0.4; mean: 0.46 *vs.* 0.55; ***Fig. 4G***), suggesting that in *DDR1*-overexpressing GC cells, YAP tends to translocate into the nucleus (***Supplementary Fig. 5***, available online). Using Kendall's tau and Spearman's rho rank correlation coefficients, we demonstrated that both YAP nuclear intensity and total score were significantly correlated with DDR1 expression (*P* = 0.0182, *r* = 0.246 for YAP nuclear intensity and *P* = 0.0288, *r* = 0.226 for YAP nuclear score, respectively; ***Fig. 4H*** and ***Supplementary Fig. 4***), but such significant correlations were not observed between DDR1 and cytoplasmic YAP. Together, these results suggest that DDR1 contributes to YAP dephosphorylation, nuclear translocation, and consequent activation of YAP-TEAD target transcription in both GC cells and tissues.

**Figure 4:**
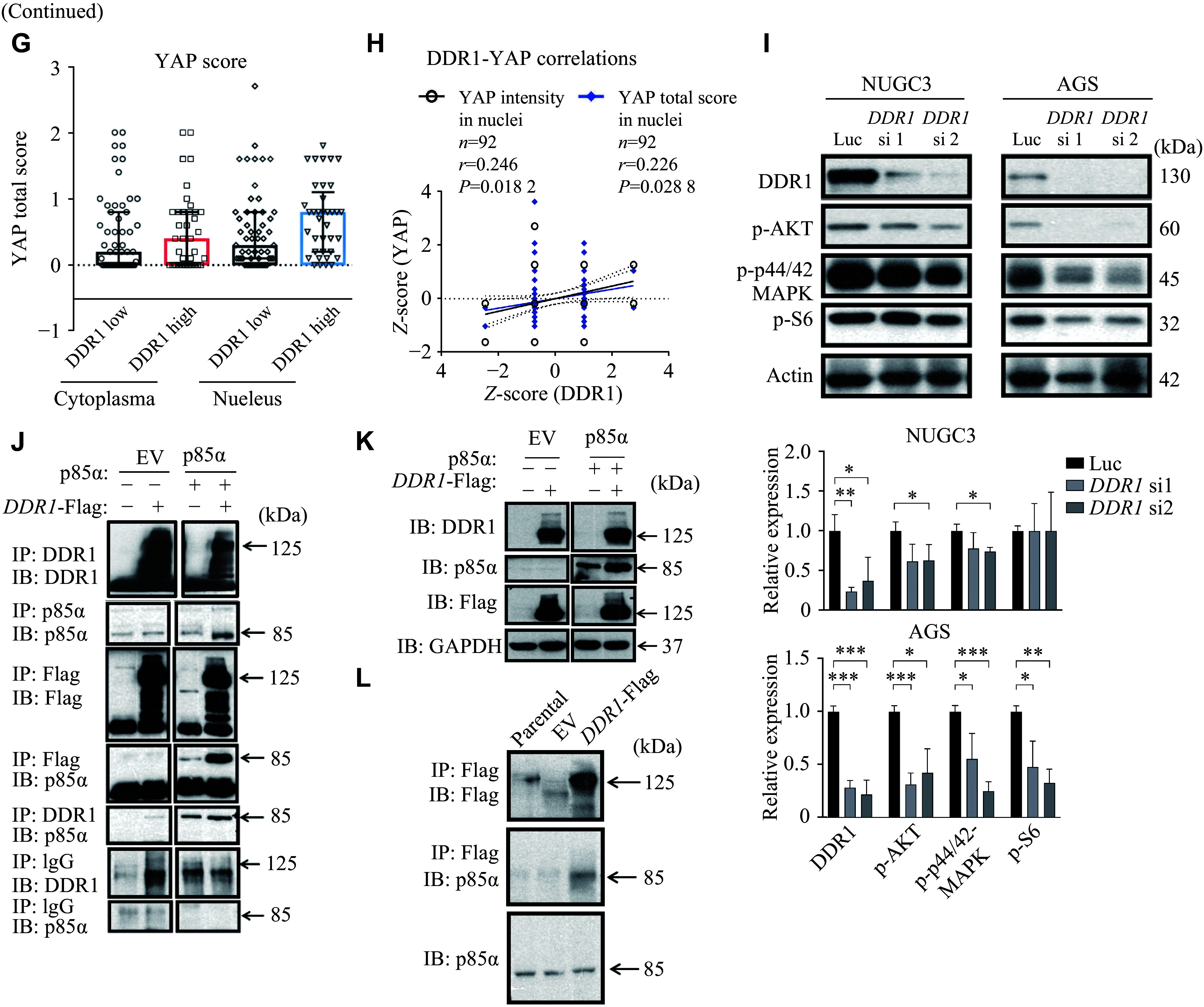
DDR1 negatively regulated the Hippo pathway and activated YAP-TEAD target gene transcription. A–C: Alterations in the expression and phosphorylation of YAP (A), 8xGTIIC-luciferase reporter (8xGTIIC-lux) activity (B), and mRNA levels of YAP-TEAD target genes (C) in NUGC3 cells receiving treatment of *DDR1* siRNAs or non-specific siRNA (NS). D: The expression and phosphorylation of YAP in *DDR1* overexpressing AGS cells. E and F: 8xGTIIC-luciferase reporter (8xGTIIC-lux) activity (E) and mRNA levels of YAP-TEAD target genes (F) in *DDR1* overexpressing (OE) AGS cells with or without further *DDR1* knockdown. Parental denotes the untreated original cell lines serving as baseline controls, while EV refers to cells transfected with empty viral vectors. Error bars represent mean ± standard deviation. ^*^P < 0.05, ^**^P < 0.01, and ^***^P < 0.001 compared the 8xGTIIC-lux activity or *DDR1* transcription with the control (NS) or parental groups by Student's *t*-test. ^ ###/+++^*P* < 0.001 compared the *CTGF*/*CYR6*1 transcriptions with the control (NS) or *DDR1* wildtype (WT) groups by Student's *t*-test. G: Distribution of cytoplasmic and nuclear YAP total score in 92 gastric cancer samples with high or low expression of DDR1. H: Correlation analysis between DDR1 expression and nuclear YAP intensity and total score. I: NUGC3 and AGS cells were transfected with either control or *DDR1*-specific siRNAs. Protein levels of p-Akt, p-p44/42-MAPK, and p-S6 in whole cell lysates were determined by Western blotting. J–K: DDR1 and p85α were co-expressed in 293T cells, and the total cell lysates were subjected to immunoprecipitation (J) and immunoblotting (K) with specific DDR1/p85α/Flag or non-specific antibody (IgG). L: Only endogenous expression of p85α was blotted after immunoprecipitation with DDR1/Flag antibodies. Error bars represent mean ± standard deviation (*n* = 3). ^*^P < 0.05, ^**^P < 0.01, and ^***^P < 0.001 by Student's *t*-test.

To further investigate the link between DDR1 and YAP, we first examined the AKT and MAPK signaling pathways in GC cells because they are two well-established signaling cascades downstream of RTKs, including DDR1^[25–26]^. Indeed, *DDR1* knockdown led to reduced levels of both p-MAPK and p-AKT in NUGC3 and AGS cells (***Fig. 4I***). In 2013, Fan *et al*^[27]^ reported that EGF signaling inhibited the Hippo pathway by activating PI3K and PDK1, leading to the dissociation of the Hippo core complex. Thus, we hypothesized that DDR1 might also regulate the Hippo pathway by interfering with the formation of the PDK1-Hippo complex *via* its interaction with PI3K. One piece of evidence supporting this hypothesis is that there is a YELM-binding motif in the C-terminus of DDR1, which provides a binding site for the p85α subunit of PI3K, but the binding of DDR1 and p85α has not been demonstrated to date. Next, we studied the interaction between DDR1 and p85α in 293T cells overexpressing p85α and/or DDR1-Flag. Indeed, both DDR1 and the Flag antibody successfully pulled down p85α (***Fig. 4J*** and ***4K***) in a co-immunoprecipitation system. In addition, immunoprecipitation of exogenous DDR1 also co-precipitated endogenous p85α (***Fig. 4L***). Based on the above evidence, we proposed that DDR1 may negatively regulate the Hippo signaling pathway *via* binding to the p85α subunit, activating the PI3K pathway, and consequently dissociating the Hippo core complex.

### DDR1-specific inhibitors blocked the proliferation and growth of multiple GC cells

Currently, only a few molecules that selectively target DDR1 are available for research, and we used three of them in this study, namely, 7rh^[10]^ (***Fig. 5A***), DDR1-IN-1^[11]^ (***Fig. 5B***), and KST9046^[28]^ to study their effects on GC inhibition. First, the anti-proliferative activity of each compound was evaluated in HFE145 and five GC cell lines. The responses to the three inhibitors varied among different cell lines. The average IC_50_ for DDR1-IN-1 is 2.549 μmol/L, which was comparable to 7rh (3.627 μmol/L) (***Fig. 5A*** and ***5B***), but the colony formation assay revealed that the sustained anti-tumor activity of DDR1-IN-1 monotherapy was substantially attenuated compared with 7rh over time, suggesting a divergence in durability of effects despite comparable initial efficacy (***Fig. 5C***). Because the average IC50 for KST9046 was higher than the other two (above 5 μmol/L), this compound was omitted from further investigation (Data not shown). Next, the effect of target inhibition of 7rh and DDR1-IN-1 in GC cells was examined by probing for the phosphorylation status of DDR1. Both compounds effectively inhibited DDR1 phosphorylation (***Fig. 5D***). In addition, 7rh but not DDR1-IN-1 treatment led to a reduction in the DDR1 expression level, which had been previously reported by Gao *et al*^[10]^.

**Figure 5:**
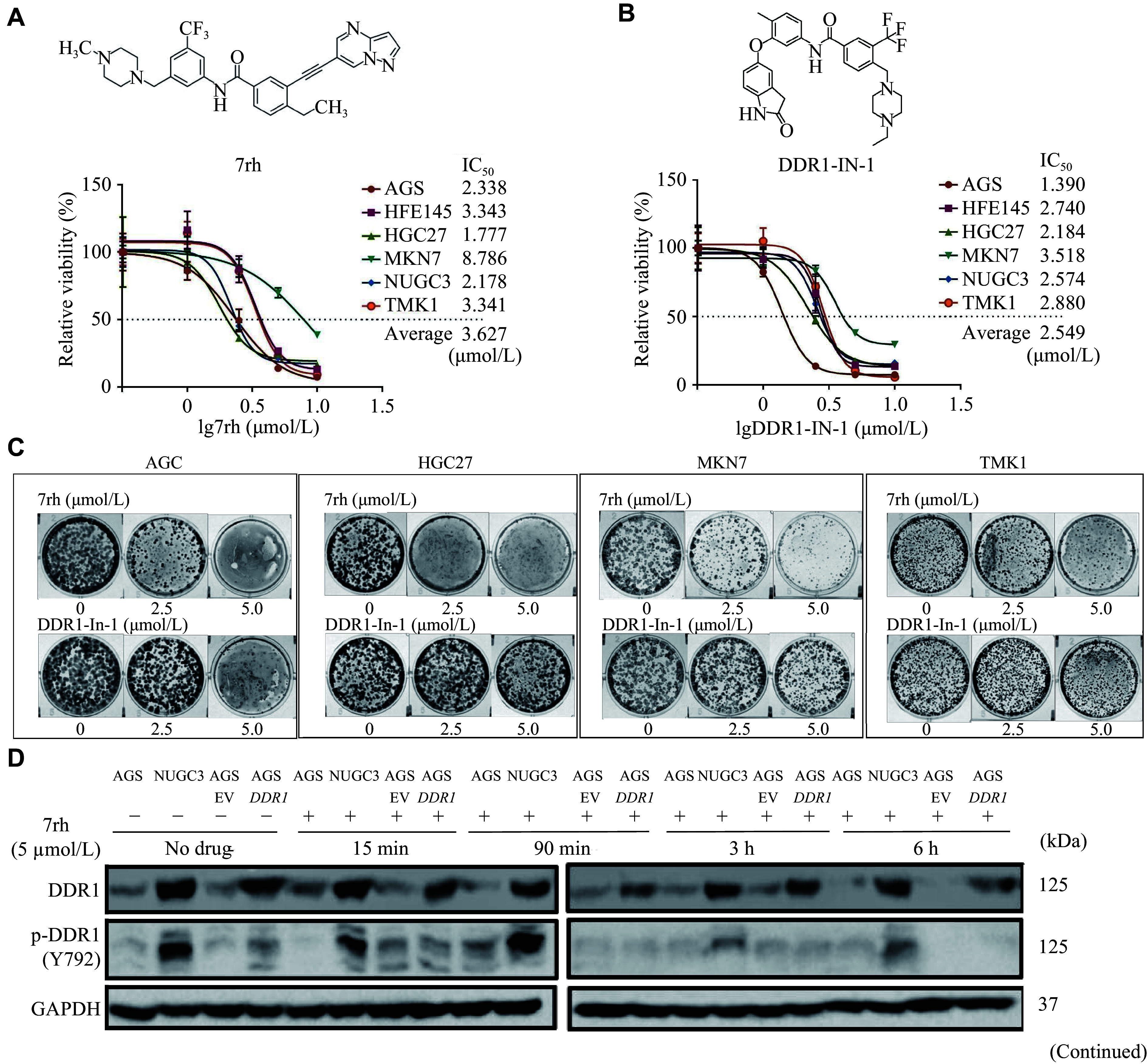


**Figure 5:**
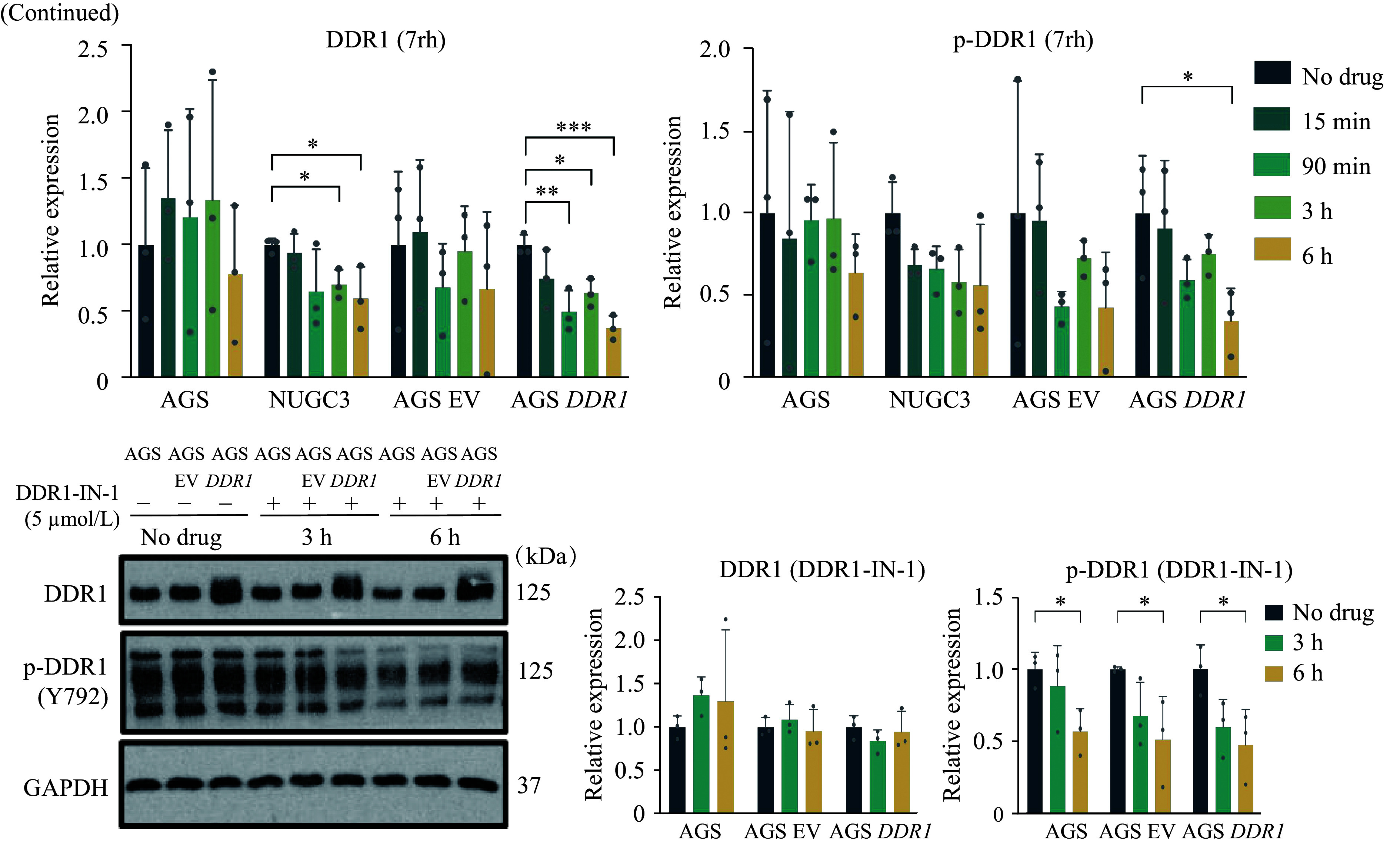
Anti-proliferative effect of DDR1-specific inhibitors on gastric cancer (GC) cells. A–B: Anti-proliferation curves and IC_50_ of six GC cell lines that received DDR1-specific inhibitor 7rh (A) and DDR1-IN-1 (B). Cells were exposed to various concentrations of DDR1 inhibitors for three days. The structures of these two compounds are shown above. Error bars represent mean ± standard deviation. C: Colony formation assay of multiple GC cells untreated or treated with 0, 2.5, and 5 µmol/L of 7rh or DDR1-IN-1. Cells were fixed and stained once the control wells reached confluency. D: Suppressive effects of 7rh and DDR1-IN-1 on DDR1 expression and phosphorylation in GC cells were examined by immunoblotting with the indicated antibodies. Error bars represent mean ± standard deviation (*n* = 3). ^*^P < 0.05, ^**^P < 0.01, and ^***^P < 0.001 by Student's *t*-test.

### GC cells overexpressing *DDR1* were sensitive to DDR1-selective inhibitors both *in vitro* and *in vivo*

**Figure 6:**
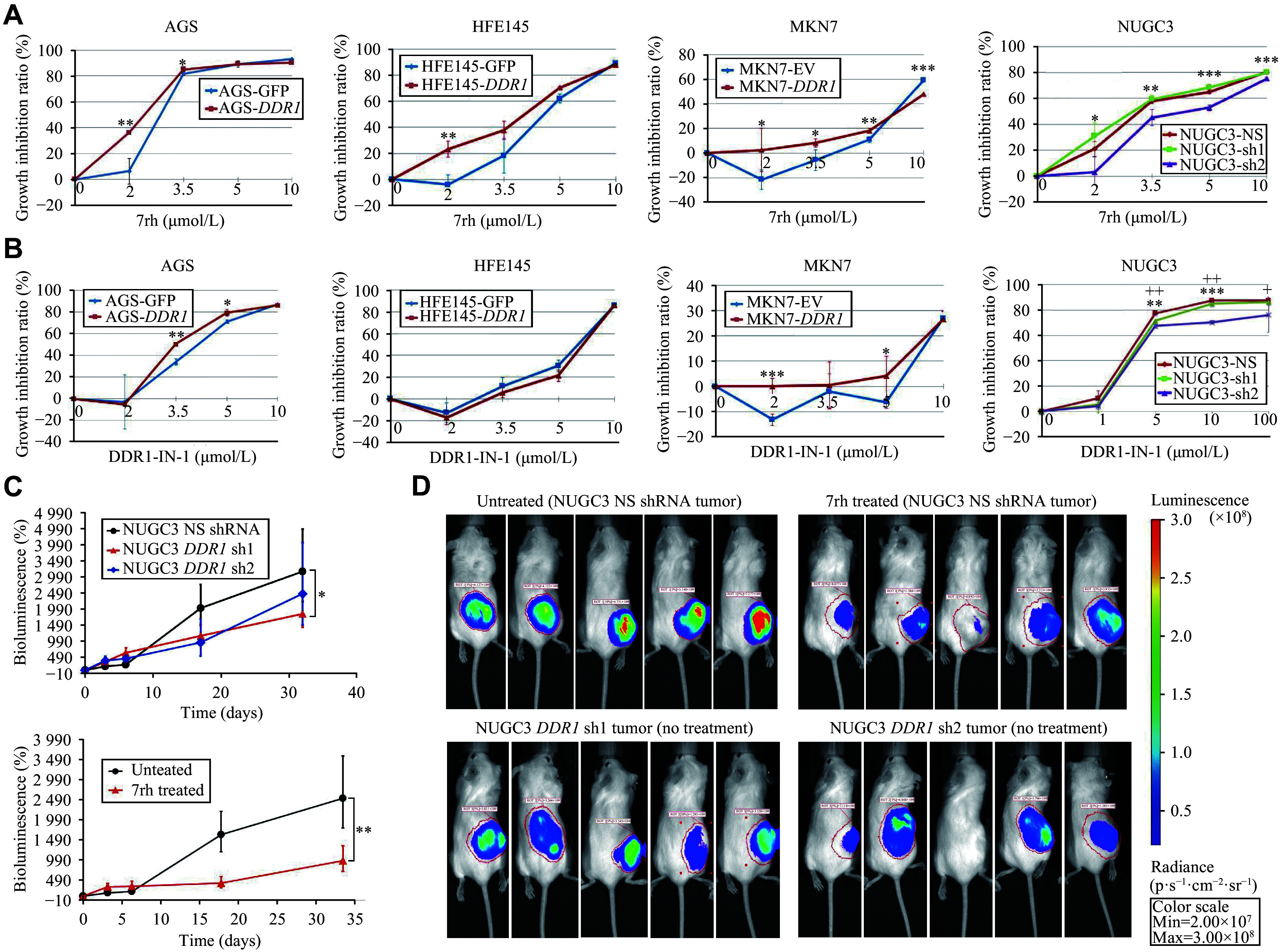
The efficacy of DDR1-selective inhibitors 7rh and DDR1-IN-1 on *in vitro* and *in vivo* *DDR1* overexpressing gastric cancer models. *DDR1* was overexpressed in AGS, HFE145, and MKN7 cells (DDR1 low or moderate), while NUGC3 cells had *DDR1* knockdown with two *DDR1* shRNAs (sh1 and sh2) or a non-silencing (NS) shRNA as the control. Two sets of cells were then treated with DDR1-specific inhibitor 7rh (A) or DDR1-IN-1 (B) for three days, respectively, at the indicated concentrations. Error bars represent mean ± standard deviation (*n* = 5). ^*^P < 0.05, ^**^P < 0.01, and ^***^P < 0.001 compared the growth inhibition ratio of *DDR1* overexpressed cell lines or NUGC3_sh2 with the control group. ^+^*P* < 0.05 and ^++^*P* < 0.01 compared the growth inhibition ratio of NUGC3_sh1 with the control group. C–D: *In vivo* drug efficacy study of 7rh was performed using SCID mice carrying NUGC3 *DDR1* knockdown or control (non-silencing [NS] NUGC3) xenograft tumor. Mice were administered an oral formulation of 7rh *via* oral gavage at 25 mg/kg or no drug (*n* = 6 per group). The bioluminescence intensity curve indicates tumor growth in response to 7rh (C). Representative images of tumor bioluminescence from each group (D). Error bars represent mean ± standard error of the mean. ^*^P < 0.05 and ^**^P < 0.01 by Student's *t*-test.

The data above demonstrated the antiproliferative activities of DDR1 inhibitors in blocking the proliferation of GC cells. A pertinent question is whether the level of DDR1 as a drug target could determine cellular responses to the drug. A few isogenic cell lines stably overexpressing *DDR1* were therefore generated to address this question. As shown in ***Fig. 6A*** and ***6B***, GC cells with *DDR1* overexpression were more sensitive to the inhibitors than those expressing low levels of *DDR1*. In another cellular model, when NUGC3 cells (DDR1 high) with a non-silencing control shRNA or *DDR1* being stably knocked down were treated with DDR1 inhibitors, the latter became less sensitive to both 7rh and DDR1-IN-1 than control cells. Additionally, a multi-targeted kinase inhibitor, imatinib, also inhibited the activity of DDR1, and overexpression of *DDR1* rendered the GC cells more sensitive to imatinib than control cells. Similarly, *DDR1*-silenced NUGC3 cells were less responsive to imatinib than control cells (***Supplementary Fig. 6***, available online).

Lastly, we studied the efficacy of 7rh in a GC xenograft model to determine whether the *in vitro* observations could be recapitulated *in vivo*. As shown in ***Fig. 6C*** upper, stable knockdown of *DDR1* by two specific shRNAs caused retarded growth of NUGC3 xenograft tumor (measured as the ratio of bioluminescence increase over time zero). Consistent with this result, pharmacological inhibition of DDR1 by 7rh (25 mg/kg q.d. for two weeks) caused a significant suppression of the growth curve of NUGC3 xenograft tumor relative to the control group that received vehicle solution only (***Fig. 6C*** lower). Representative images of tumor bioluminescence from each group are shown in ***Fig. 6D***. Overall, these results underscore *DDR1*'s pivotal role in GC progression and suggest that gastric tumors with *DDR1* overexpression may benefit from treatment with DDR1-selective inhibitors.

## Discussion

The present study identifies DDR1 as a potential drug target and tumor biomarker through an iTRAQ-based phosphoproteomic screen. *DDR1* overexpression was highly prevalent in GC and associated with shorter OS and FP. Using *in vitro* models, we demonstrated that DDR1 drove tumorigenesis by activating the PI3K-AKT signaling pathway and YAP-TEAD target gene expression. Furthermore, selective DDR1 inhibitors were demonstrated to be capable of blocking the rapid growth of DDR1-positive gastric tumors *in vivo*. Our tissue microarray data from more than 200 patients showed that DDR1 was overexpressed in approximately half of all GCs (41% for intestinal and 48% for diffuse types) and in more than half of early-stage tumors (50% for intestinal and 68% for diffuse types). Thus, a substantial subgroup of GC patients can benefit from DDR1-targeted therapy.

In this study, we focused on DDR1 overexpression rather than mutation. The mutation rate of *DDR1* is only 2.93%, according to the online database COSMIC (*n* = 409)^[29]^. Not only is the mutation rate of *DDR1* low in GC, but it is also unknown whether these mutations contribute to tumorigenesis. Overexpression of wild-type *DDR1* is commonly observed in various cancers, indicating that it represents the primary oncogenic alteration of *DDR1*. For example, in 146 NSCLC tissue samples, *DDR1* was markedly upregulated and significantly associated with overall and disease-free survival, whereas *DDR2* expression was downregulated and not prognostic^[30]^.

Recent research highlights the significance of DDR1 in cancer, though its precise role in cancer progression is still debated. Firstly, DDR1 could act as both a tumor suppressor or promoter depending on cellular contexts. Although it is generally believed that DDR1 is more likely to act as a suppressor of oncogenic events/signaling in non-malignant cells, and as a cancer promoter in malignant cells, such a role could be reversed^[22]^. Secondly, DDR1 regulates a myriad of downstream signaling pathways in different types of cancer, such as the pro-survival Ras/Raf/ERK and PI3K/Akt pathways in human breast and colon cancer cells^[26]^; the FAK-p130CAS/JNK pathway, which results in mesenchymal transition-like cell scattering in pancreatic cancer cells^[31]^; and the SHP2 signaling pathway, which inhibits STAT1 and STAT3 tyrosine phosphorylation in diverse cancer cells^[32]^. It is likely that DDR1 is connected to a complex and dynamic signaling network, and the signaling pathway that is dominantly activated within this network is highly context-dependent and cancer type-specific. In the current study, we observed that *DDR1* overexpression activated the AKT and YAP-TEAD signaling axis, which drove the oncogenic transcription program associated with an increase in cancer cell proliferation. Additionally, YAP/TAZ activation may also be mediated through other pathways, such as the cilium-dependent pathway involving AMPK and SIRT1, which is independent of the Hippo pathway regulation^[33]^. While the current study elucidates the functional association between DDR1 and YAP-TEAD signaling, these findings collectively indicate potential heterogeneity in the activation mechanisms of YAP/TEAD complexes. Further investigations are required to elucidate the precise molecular hierarchy regulating their activation, which will be critical for translating these mechanistic insights into clinically actionable therapeutic strategies.

In the *in vivo* drug efficacy study, the DDR1 inhibitor 7rh exhibited potent inhibitory effects on the growth of high *DDR1*-expressing gastric tumors even as a single agent.

Compared with broad-spectrum Bcr-Abl inhibitors like imatinib, nilotinib, and dasatinib, which target multiple kinases, a selective DDR1 inhibitor is expected to cause less toxicity^[34]^ and be especially efficacious in treating *DDR1*-overexpressing cells. Furthermore, combination therapy may further improve the anti-tumor activity of DDR1 inhibitors. It has been shown by Kim *et al*^[11]^ that when DDR1 inhibitor DDR1-IN-1 (which is less potent than 7rh in this study) was combined with AZD8055, an mTOR inhibitor, the antiproliferative activity was substantially potentiated in colorectal cancer cells. Thus, one feasible application of the DDR1 inhibitors is to combine them with conventional chemotherapy or other targeted agents to provide a novel solution for treating refractory DDR1-positive GC.

In summary, overexpression of the DDR1 receptor tyrosine kinase has a high penetrance in GC, causing accelerated proliferation of GC cells *via* Hippo pathway inhibition and consequent activation of YAP-TEAD target gene expression. Moreover, DDR1-selective small-molecule inhibitors displayed potent GC tumor inhibitory effects, particularly in *DDR1*-overexpressing cells. While the current findings remain preclinical and require extensive validation through both *in vitro* and *in vivo* studies, it is important to evaluate the efficacy of these small molecules as a monotherapy or in combination therapy in the management of GC in future clinical trials.

## SUPPLEMENTARY DATA

Supplementary data to this article can be found online.
