# Supplementary material for: Overexpression of DDR1 contributes to gastric cancer progression by inhibiting the Hippo pathway
Source: J Biomed Res. 2025 Jun 3;39(5):500–14. doi: 10.7555/JBR.39.20250198 (PMC12481679; doi:10.7555/JBR.39.20250198)
Supplement: Supplementary file 1 — Supplementary data to this article can be found online. [file jbr-39-5-500-Supplementary.pdf]

Available online at www.jbr-pub.org.cn

Open Access at PubMed Central

Journal of Biomedical Research, 2025 39(5): 1–6

JBR

Supplementary Data

Overexpression of *DDR1* contributes to gastric cancer progression by inhibiting the Hippo pathway

Haiying Han[1,△], Tianqi Shen[2,△], Tingting Zhou[2,△], Yixuan Yang[3], Weiyi Toy[3], Yin Yin Choo[3], Fan Lin[2,✉], Yoon Pin Lim[3,4,5,✉]

[1]*Department of Nursing, School of Medicine, Hangzhou City University, Hangzhou, Zhejiang 310000, China;*
[2]*Department of Cell Biology, School of Basic Medical Sciences; Institute for Brain Tumors & Key Laboratory of Rare Metabolic Diseases; The Affiliated Cancer Hospital, Nanjing Medical University, Nanjing, Jiangsu 211166, China;*
[3]*Department of Cancer Biology and Innovation, Guoke Ningbo Life and Health Industry Research Institute, Ningbo, Zhejiang 315000, China;*
[4]*Department of Biochemistry, Yong Loo Lin School of Medicine, National University of Singapore, Singapore 117545, Singapore;*
[5]*NUS Graduate School of Integrative Sciences and Technology, Singapore 117456, Singapore.*

Supplementary methods

Reagents

Anti-DDR11 (D1G6) antibody (Cat. #5583), anti-phospho-DDR1 (Tyr792) rabbit polyclonal antibody (Cat. #5583), anti-YAP (Cat. #14074), anti-phospho-YAP (Ser127) (Cat. #13008), anti-phospho-pAKT (Ser473) (Cat. #4060), anti-phospho-S6 (Cat. #2211), Hippo Signaling Antibody Sampler Kit (Cat. #8579), anti-V5 (Cat. # 13202), anti-FLAG (Cat. ##14793), anti-GADPH (Cat. #2118), and β-Actin (Cat. #4970) were purchased from Cell Signaling Technology (Danvers, MA, USA). Anti-mouse and anti-rabbit peroxidase (HRP) conjugated secondary antibodies were purchased from Thermo Fisher Scientific Pierce (Rockford, USA). Anti-DDR1 (Cat. #sc-532, Santa Cruz, CA, USA) rabbit polyclonal antibody was used for immunohistochemistry (IHC) staining.

DDR1-IN-1 dihydrochloride was purchased from Tocris (Bristol, UK), and KST9046 was obtained from Dr. Elkamhawy and Dr. Roh.

pENTR4-FLAG, pLenti CMV GFP Puro, pLenti CMV Puro DEST, and 8xGTIIC used for plasmid construction or virus packaging, *etc*., were gifts from William Sellers (Addgene plasmid Cat. #10785), Eric Campeau (Addgene plasmid Cat. #17448, Cat. #17452), and Stefano Piccolo (Addgene plasmid Cat. #34615). Non-silencing and three *DDR1* pGIPZ lentiviral shRNA clones (clone ID: V3LHS_392923, V2LHS_84433, and V2LHS_202770) were provided as glycerol stock (Thermo Fisher Scientific Pierce).

Mass spectrometry

Peptides labeled with isobaric tags for relative and absolute quantitation (iTRAQ) were dissolved in 300 μL of 8 mol/L urea and 1% Pharmalyte (Amersham Biosciences, England) to rehydrate IPG strips (18 cm long, pH 3–10, Amersham Biosciences) for 14 h at 30 volts. Peptides were concentrated sequentially at 500 V for 1 h, 1 000 V for 1 h, and 8 000 V for 8.5 h, resulting in a total of 68 kV·h using the IPGphor system (Amersham Biosciences). The strips were removed and swiftly sliced into 36 pieces, each 0.5 cm in length. Peptides were extracted by soaking the gel pieces in 100 μL of a solution containing 2% acetonitrile and 0.1% formic acid for 1 h. Using a vacuum concentrator, fractions were lyophilized and then purified using a C18 Discovery DSC-18 SPE column (100 mg capacity, Supelco, Sigma-Aldrich). Before the mass spectrometric

△These authors contributed equally to this work.

✉Corresponding authors: Fan Lin, XueHai Building A111, Nanjing Medical University, 101 Longmian Avenue, Nanjing, Jiangsu 211166, China. E-mail: linfee@me.com; Yoon Pin Lim, Block B2, New Materials Innovation Center, Juxian Street, Ningbo, Zhejiang 315000, China. E-mail: linyunbin@ucas.ac.cn.

Received: 09 May 2025; Revised: 22 May 2025; Accepted: 28 May 2025; Published online: 03 June 2025

CLC number: R735.2, Document code: A

The authors reported no conflict of interests.

This is an open access article under the Creative Commons Attribution (CC BY 4.0) license, which permits others to distribute, remix, adapt and build upon this work, for commercial use, provided the original work is properly cited.

© 2025 by Journal of Biomedical Research.

https://doi.org/10.7555/JBR.39.20250198

analysis, the cleaned fractions were stored at –20 ℃ after being lyophilized again.

Every purified peptide fraction was resuspended in 20 μL of Buffer A, which contained 0.1% formic acid in 2% acetonitrile, and 10 μL of this sample was injected into the nano-LC-ESI-MS/MS system. Mass spectrometry was performed using a QStar Elite Hybrid ESI Quadrupole time-of-flight tandem mass spectrometer and ESI-Q-TOF-MS/MS (MDS-Sciex, Concord, Canada; Applied Biosystems, Framingham, USA) coupled to an online capillary liquid chromatography system (Dionex Ultimate 3 000, Amsterdam, The Netherlands). The peptide blend was divided using a PepMap C-18 RP capillary column (flow rate: 0.3 μL/min, Dionex). A gradient lasting 125 min was employed, beginning with 96% Buffer A and 4% Buffer B (0.1% formic acid in 98% acetonitrile) for the first 3 min. Subsequently, the chromatographic separation employed a segmented elution profile: initial 7-min ramp (4%→10% Buffer B), followed by 55-min development phase (10%→35% B), concluding with 25-min steep gradient (35%→100% Buffer B). Post-separation conditioning comprised 15-min isocratic elution at 100% Buffer B, followed by 20-min re-equilibration at 96% Buffer A. Mass spectrometric detection operated in positive polarity mode with scanning parameters configured as 300–1 800 m/z. MS/MS acquisition employed a 2-second signal integration window. The two highest-intensity precursors meeting the 20-count threshold underwent MS/MS analysis, followed by dynamic exclusion (30 s duration) implemented under 50 mDa mass accuracy constraints. iTRAQ sample proteins were analyzed using ProteinPilot software (Applied Biosystems, MDS-Sciex). The search was conducted using the International Protein Index (IPI) human database, which contained 72 155 sequences (version 3.41). Single peptide hits were not included in further analysis.

Transfection

Cells were plated at 65%–75% confluence with complete medium one day prior to transfection for knockdown and overexpression studies, and then transfected with 200 nmol/L siRNA and 10 μL jetPRIME reagent (Polyplus Transfection Inc.)

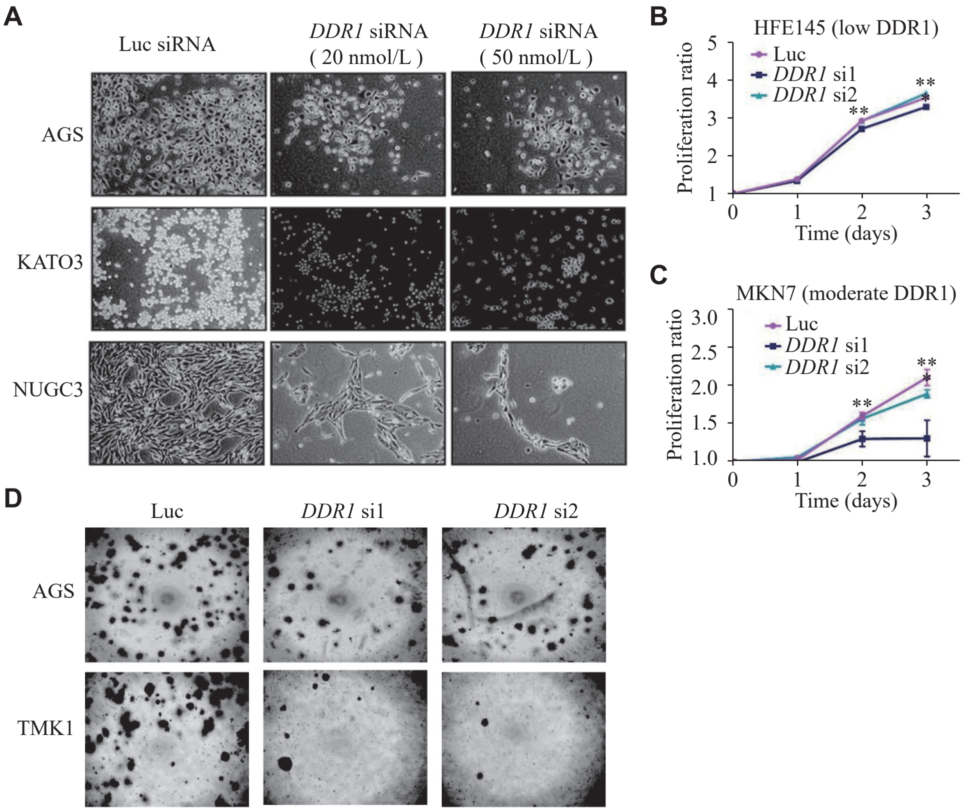

Supplementary Fig. 1 ***DDR1*** **knockdown reduced the invasion and migration capacities of gastric cancer (GC) cells.** A–C: Representative microscopic images of AGS, KATO3 and NUGC3 cells and proliferation curves of HFE145 and MKN7 cells treated with control or two *DDR1*-specific siRNA (10× magnification). Error bars represent mean ± standard deviation (n = 5). $^{**}P$ < 0.01. D: Representative microscopic images of AGS, KATO3 and NUGC3 cells invaded across the transwell membranes after treatment with control or *DDR1*-specific siRNA (10× magnification, stained with crystal violet).

following the manufacturer's instructions. Cells were harvested at 48 h post-transfection, with mock transfections and non-specific siRNA duplexes serving as negative controls. Cells underwent treatment for 48 h to 72 h to ensure optimal knockdown, followed by harvesting for Western blotting or application in functional tests.

Lentivirus production and transduction

293T cells were transiently co-transfected with either the *DDR1*-targeting lentiviral vector (shRNA-pLenti-CMV-Puro/Hygro for knockdown) or the *DDR1*-Flag expression construct (pLenti-CMV-Puro for overexpression), combined with envelope/packaging plasmids pMDLg/pRRE, pRSV-Rev, and pCMV-VSVG at a mass ratio of 5∶2∶2∶1, followed by replacing the medium with complete medium 6–8 h post-transfection. The virus supernatant was collected 48 h after transfection, then clarified by centrifugation and filtered through a 0.45 µm filter. Cells were plated at 60%–70% confluence one day before lentivirus transfection. The polybrene-supplemented (4 µg/mL, Sigma) viral supernatant was added to the cells, followed by centrifugation at 1 000 *g* for 120 min at ambient temperature. After centrifugation, cell-virus complexes were maintained in co-culture for 24 h prior to complete medium renewal. Forty-eight hours after infection, the cells were used either for drug selection to generate stable cell lines or directly for additional experiments.

Immunohistochemistry

Tissue microarrays and individual sections were heated in a 60 ℃ oven, then dewaxed using three xylene changes and rehydrated through graded ethanol concentrations (100%, 95%, 70%) before a final rinse in double-distilled H_2O. Heat-mediated epitope retrieval was performed using Dako buffer (pH 6.0, 95 ℃, 40 min). Tissue sections sequentially underwent endogenous peroxidase inhibition (3% H_2O_2, 10 min) and protein blocking (5% BSA, 30 min, room temperature). DDR1-specific primary antibody (1∶500 dilution) was applied to tissue sections at ambient temperature (25 ± 2 ℃, 60 min). DAB chromogenic development (2 min) was performed using Envision+/HRP detection kits (Dako Cytomation, Denmark). All tissue sections underwent Gill's nuclear staining (1 min), graded alcohol

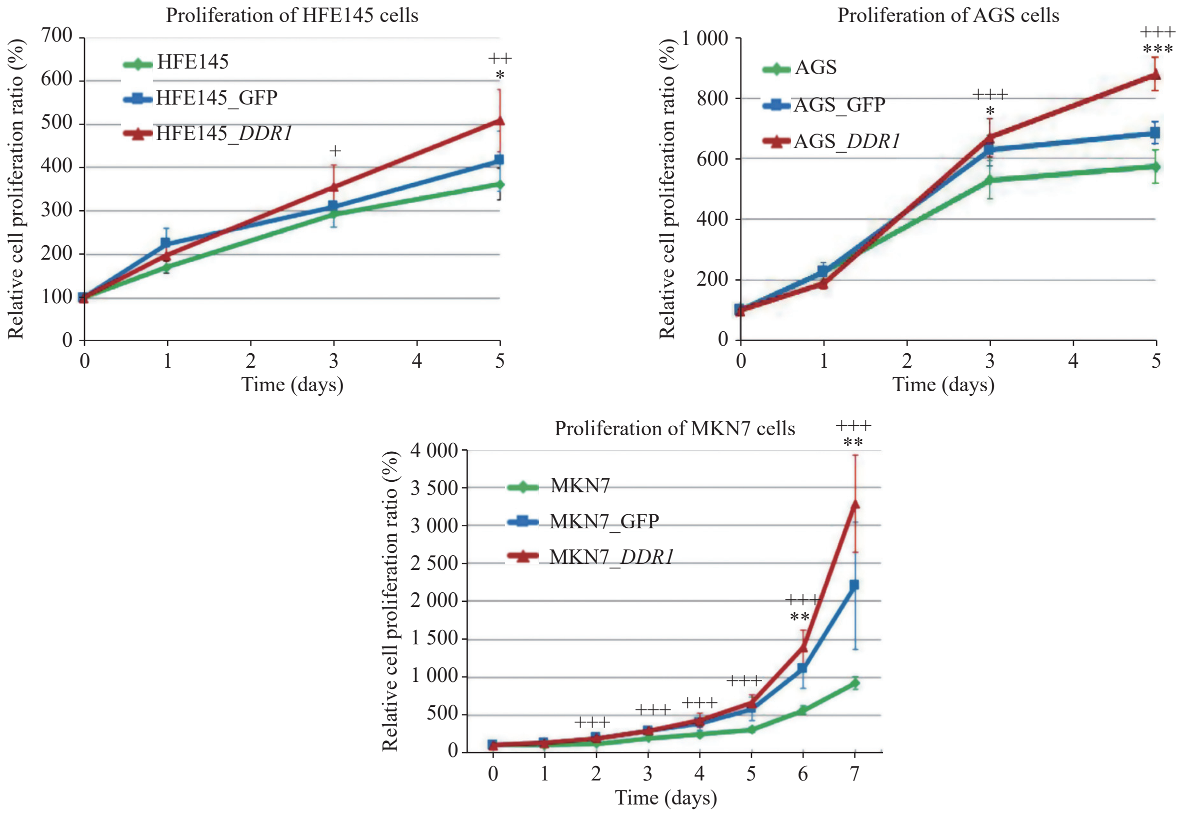

***Supplementary Fig. 2* Proliferation curves of three gastric cell lines overexpressing *DDR1*.** Error bars represent mean ± standard deviation (n = 5). $^{*}P$ < 0.05, $^{**}P$ < 0.01, $^{***}P$ < 0.001 compared the relative cell proliferation ratio of vector control cell lines with the homologous parental cell line. ^{+}P < 0.05, ^{++}P < 0.01 and ^{+++}P < 0.001 compared the relative cell proliferation ratio of *DDR1* overexpressed cell lines with homologous parental cell lines.

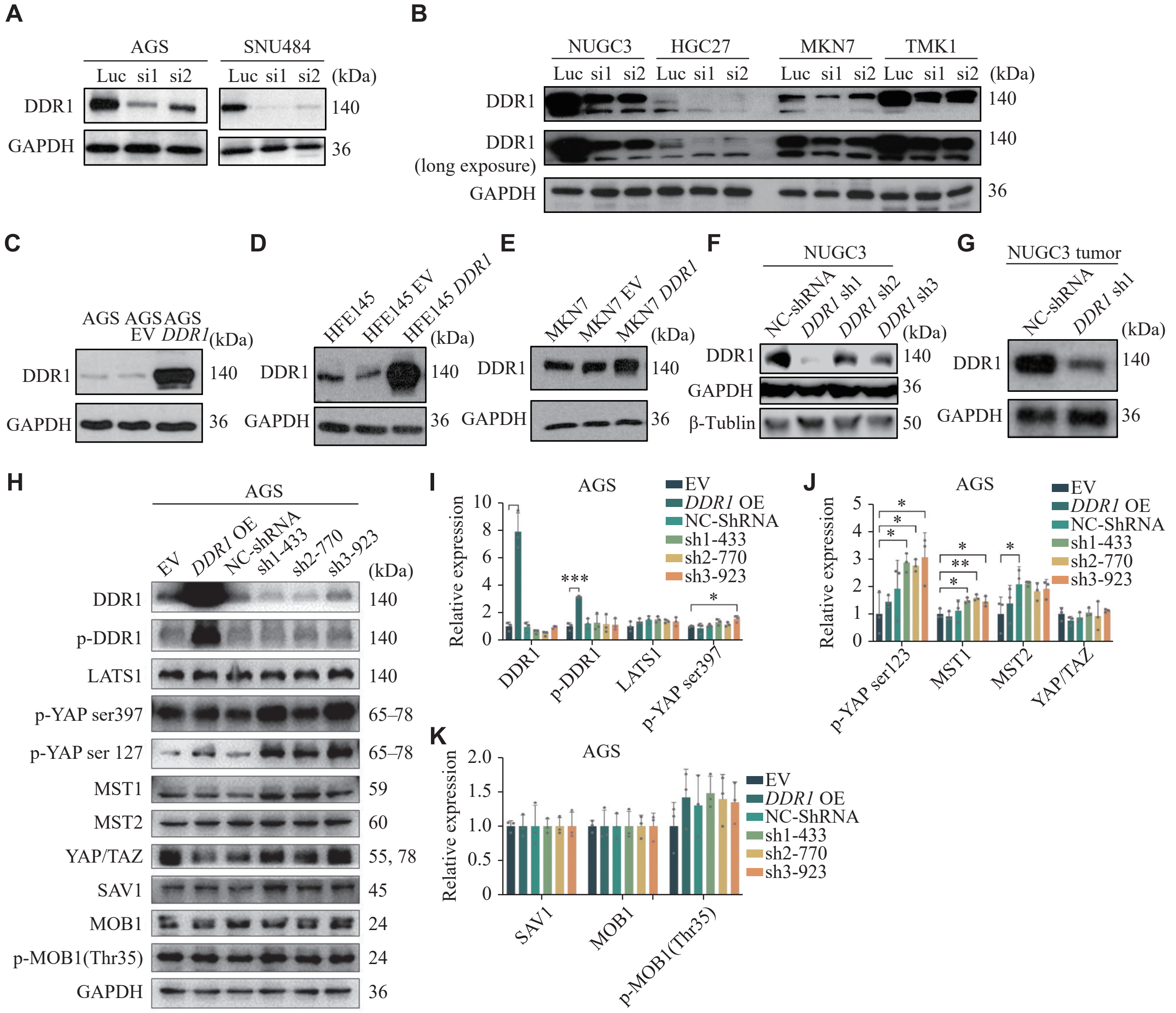

Supplementary Fig. 3 **Western blotting verification of DDR1 expression in various *DDR1* overexpressed or knockdown gastric cancer cell lines.** A–B: Two different *DDR1* siRNAs, si1 and si2, exhibited activities to silence *DDR1* in various GC cell lines, including AGS and SNU484 (A), and NUGC3, HGC27, MKN7, TMK1 (B), used in experiments of ***Figs. 3*** and ***4***. C–E: Verification of *DDR1* overexpression in AGS (C), HFE145 (D), and MKN7 (E) cell lines, used in experiments in ***Figs. 4*** and ***6*** and ***Supplementary Figs. 2*** and ***6***. F–G: Verification of *DDR1* silencing effects with *DDR1*-specific shRNA, used in ***Fig. 6*** and ***Supplementary Fig. 6***. H–K: Western blotting verification of associated alterations in Hippo pathway components in *DDR1* overexpressed and knockdown AGS cell line. Error bars represent mean ± standard deviation ($n = 3$). $^{*}P < 0.05$, $^{**}P < 0.01$, and $^{***}P < 0.001$.

processing, and optical clearing for bright-field microscopy. To ensure consistency, all IHC stains were evaluated in a blinded manner by the same certified pathologist. A percentage was noted for the proportion of stained tumor cells. H-scoring employed a dual-factor algorithm: staining intensity (0–3 scale) multiplied by positive area percentage. Using this formula, a section with 50% positively stained area demonstrating +2 intensity would generate a histochemical product value of 1. All analyses were conducted using SPSS software.

Anchorage-independent growth detection

Agar suspension cultures were quantitatively analyzed to measure anchorage-independent growth capacity. Cells were suspended in 0.35% agarose-containing complete medium and layered onto 0.6% agarose-coated 6-well plates. After 14–21 days of culture, colonies (> 50 μm diameter) were stained with crystal violet and quantified using ImageJ software.

Matrigel invasion detection

A dual-compartment system was established with serum-restricted cells in FBS-depleted matrix occupying the apical compartment, juxtaposed against a basolateral reservoir containing a 10% serum protein chemoattraction gradient. Transwell membranes underwent 24 h incubation followed by mechanical

- Total 92 patients analyzed
- IHC DDR1 expression score=0, 1, 2, 3
- Nuclear YAP intensity score=0, 1, 2, 3
- Nuclear YAP percentage=0%–100%

Nuclear YAP intensity correlations

			DDR1	Nuclear_YAP intensity
Kendall's tau_b	DDR1	Correlation coefficien	1.000	0.230
		Sig. (2-tailed)	.	0.019
		N	92	92
	Nuclear_YAP_intensity	Correlation coefficien	0.230	1.000
		Sig. (2-tailed)	0.019	.
		N	92	92
Spearman's rho	DDR1	Correlation coefficien	1.000	0.246
		Sig. (2-tailed)	.	0.018
		N	92	92
	Nuclear_YAP_intensity	Correlation coefficien	0.246	1.000
		Sig. (2-tailed)	0.018	.
		N	92	92

*Correlation is significant at the 0.05 level (2-tailed).

Nuclear YAP total score = YAP nuclear intensity x percentage correlations

			DDR1	Nuclear_YAP
Kendall's tau_b	DDR1	Correlation coefficient	1.000	0.192
		Sig. (2-tailed)	.	0.030
		N	92	92
	Nuclear_YAP	Correlation coefficient	0.192	1.000
		Sig. (2-tailed)	0.030	.
		N	92	92
Spearman's rho	DDR1	Correlation coefficient	1.000	0.228
		Sig. (2-tailed)	.	0.029
		N	92	92
	Nuclear_YAP	Correlation coefficient	0.228	1.000
		Sig. (2-tailed)	0.029	.
		N	92	92

*Correlation is significant at the 0.05 level (2-tailed).

Correlations

			DDR1	C_totalscore
Kendall's tau_b	DDR1	Correlation coefficient	1.000	0.197
		Sig. (2-tailed)	.	0.013
		N	115	115
	C_total score	Correlation coefficient	0.197	1.000
		Sig. (2-tailed)	0.013	.
		N	115	115
Spearman's rho	DDR1	Correlation coefficient	1.000	0.235
		Sig. (2-tailed)	.	0.012
		N	115	115
	C_total score	Correlation coefficient	0.235	1.000
		Sig. (2-tailed)	0.012	.
		N	115	115

*Correlation is significant at the 0.05 level (2-tailed).

Correlations

			DDR1	N_totalscore
Kendall's tau_b	DDR1	Correlation coefficient	1.000	0.268
		Sig. (2-tailed)	.	0.001
		N	115	115
	N_total score	Correlation coefficient	0.268	1.000
		Sig. (2-tailed)	0.001	.
		N	115	115
Spearman's rho	DDR1	Correlation coefficient	1.000	0.323
		Sig. (2-tailed)	.	0.000
		N	115	115
	N_total score	Correlation coefficient	0.323	1.000
		Sig. (2-tailed)	0.000	.
		N	115	115

**Correlation is significant at the 0.05 level (2-tailed).

Supplementary Fig. 4 **Correlation analysis between DDR1 expression and cytoplasmic/nuclear YAP intensity or YAP total score using Kendall's tau and Spearman's (rho) rank correlation coefficients.**

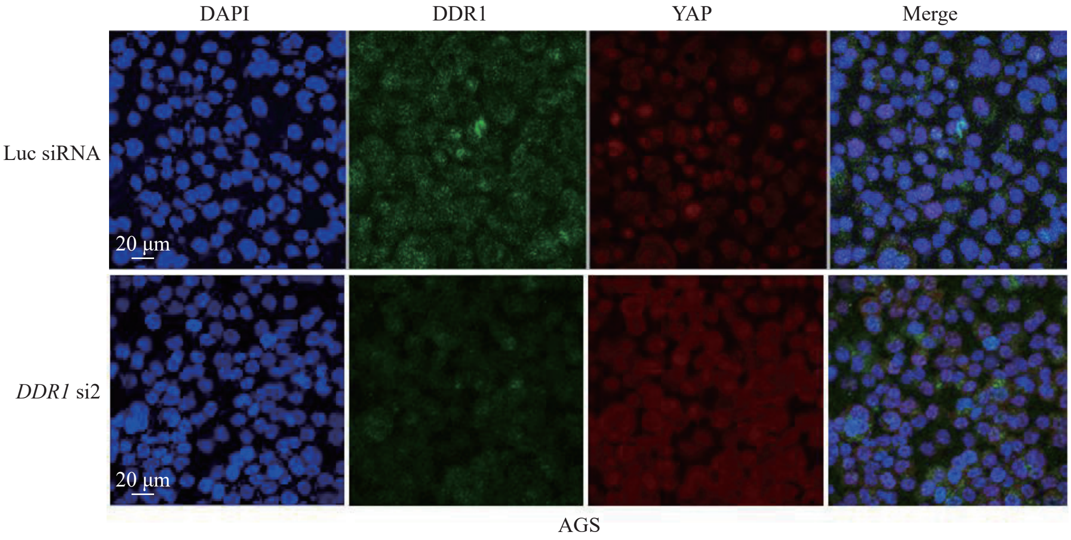

Supplementary Fig. 5 **Representative immunofluorescence micrographs illustrating DAPI nuclear counterstaining (blue), DDR1 expression patterns (green), and YAP subcellular localization (red) in AGS cells following siRNA-mediated *DDR1* knockdown.** Scale bar: 20 μm.

clearance of apical non-migratory cells. Basolateral migratory populations were methanol-fixed, 0.1% gentian violet-stained, and quantified through systematic field sampling (5 fields/membrane).

Wound healing detection

Cells were grown to 75%–85% confluence in 6-well plates. A sterile 200 μL pipette tip was used to create linear scratches. Detached cells were removed

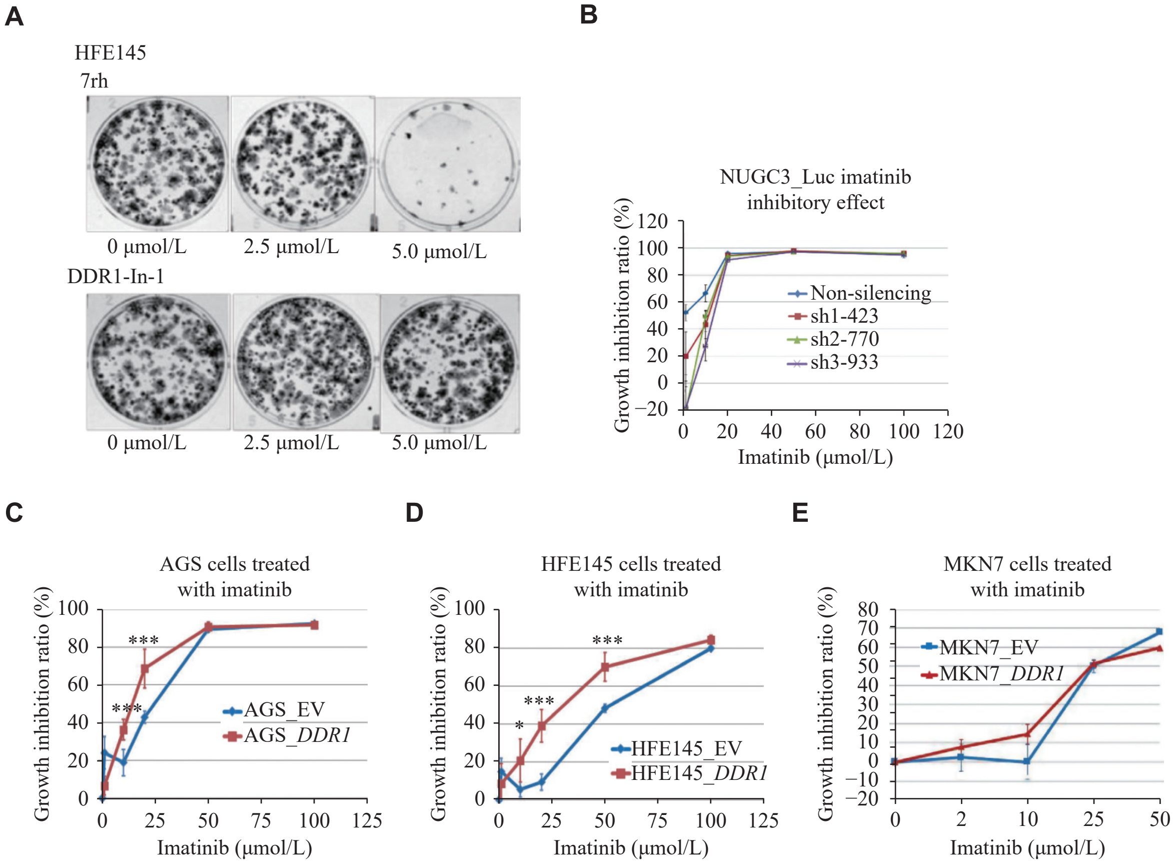

Supplementary Fig. 6 **Effects of DDR1-selective inhibitors on HFE145 cells.** A: Colony formation assay of the HFE145 cell line untreated or treated with 2.5 μmol/L and 5 μmol/L of 7rh or DDR1-IN-1. Cells were fixed and stained once the control wells reached confluency. B–E: Effects of *DDR1* overexpression or silencing on the response of gastric cancer cells to imatinib. AGS, HFE145, and MKN7 cells were infected with viruses derived from either an empty viral vector (EV) as a control or a vector containing *DDR1*-Flag. NUGC3 cells (expressing high DDR1) were transfected with either a non-silencing control or *DDR1*-specific shRNA. All cells were then treated with various doses of a general TKI inhibitor, imatinib, for 3 days. Error bars represent mean ± standard deviation (n = 5). $^{*}P < 0.05$ and $^{***}P < 0.001$.

by PBS washing. Wound closure was monitored at 0 h and 24 h using phase-contrast microscopy. Wound closure rate was calculated as: [(Initial scratch area – Final scratch area)/Initial scratch area] ×100%.
